# Bowel dysfunctions after acquired brain injury: a scoping review

**DOI:** 10.3389/fnhum.2023.1146054

**Published:** 2023-10-12

**Authors:** Matteo Zandalasini, Laura Pelizzari, Gianluca Ciardi, Donatella Giraudo, Massimo Guasconi, Stefano Paravati, Gianfranco Lamberti, Antonio Frizziero

**Affiliations:** ^1^Department of Rehabilitative Medicine, Azienda USL Piacenza, Piacenza, Italy; ^2^Department of Medicine and Surgery, University of Parma, Parma, Italy; ^3^Department of Urology, San Raffaele Hospital, Ville Turro, Milan, Italy; ^4^Dipartimento della Direzione delle Professioni Sanitarie, Azienda USL Piacenza, Piacenza, Italy

**Keywords:** bowel dysfunction, brain injury, constipation, fecal incontinence, rehabilitation

## Abstract

Bowel dysfunction is a common consequence of neurological diseases and has a major impact on the dignity and quality of life of patients. Evidence on neurogenic bowel is focused on spinal cord injury and multiple sclerosis; few studies have focused on patients with acquired brain injury (ABI). Neurogenic bowel dysfunction is related to a lifelong condition derived from central neurological disease, which further increases disability and social deprivation. The manifestations of neurogenic bowel dysfunction include fecal incontinence and constipation. Almost two out of three patients with central nervous system disorder have bowel impairment. This scoping review aims to comprehend the extent and type of evidence on bowel dysfunction after ABI and present conservative treatment. For this scoping review, the PCC (population, concept, and context) framework was used: patients with ABI and bowel dysfunction; evaluation and treatment; and intensive/extensive rehabilitation path. Ten full-text articles were included in the review. Oral laxatives are the most common treatment. The Functional Independence Measure (FIM) subscale is the most common scale used to assess neurogenic bowel disease (60%), followed by the Rome II and III criteria, and the colon transit time is used to test for constipation; however, no instrumental methods have been used for incontinence. An overlapping between incontinence and constipation, SCI and ABI increase difficulties to manage NBD. The need for a consensus between the rehabilitative and gastroenterological societies on the diagnosis and medical care of NBD.

**Systematic review registration:**

Open Science Framework on August 16, 2022 https://doi.org/10.17605/OSF.IO/NEQMA.

## 1. Introduction

Neurogenic bowel dysfunction (NBD) is defined as a loss of voluntary control of bowel function due to central nervous system (CNS) disease (Hinds et al., 1990; Edwards et al., 1992), leading to a spectrum of bowel symptoms, mainly fecal incontinence (FI; Hinds et al., 1990; Harari et al., 2003) and/or constipation (Hinds et al., 1990; Glickman and Kamm, 1996; Stocchi et al., 2000). The CNS plays a key role in gastroenteric control in terms of motor, sensory storage, and excretory functions (Camilleri, 2021). There is a complex and continuous interaction between the CNS and the enteric nervous system (ENS), nervous ganglia present within the gastroenteric wall, mainly through the sympathetic prevertebral ganglia, pelvic, and vagus nerve pathways (Furness et al., 2014). The CNS centers directly control contractile/secretive activity in the upper gastrointestinal tract, but they are also involved in lower tract motility, blood flow, electrolyte transport by reflex circuits expressed by ENS neurons, and control defecation through spinal cord lumbosacral centers (Furness et al., 2014). Brain control, along with anatomical structures and somatic and visceral peripheral innervation, ensures the physiological function of the anorectal system. Unlike the relatively well studied literature on spinal and peripheral innervation, the cerebral mechanisms regulating anorectal continence are still poorly understood (Bittorf et al., 2006). The rectum serves as a reservoir for solid and liquid feces, as well as gases produced by the small and large intestines, and it must ensure efficient emptying. The smooth and striated muscular sphincteric apparatus ensures fecal continence. The mechanisms of fecal continence and fecal evacuation are partly under the control of the same cerebral structures that ensure urinary continence (Drake et al., 2010).

The physiological sequence, under voluntary control, between filling and emptying depends on the information that reaches the brain from the periphery. Any situation that disrupts the perception, transmission or processing of this information at the cerebral level can lead to dysfunction of the lower intestinal tract (Hinds et al., 1990; Weber et al., 1990; Nakayama et al., 1997; Lotze et al., 2001; Cardozo and Staskin, 2022).

Advancements in imaging have allowed for the development of understanding regarding the cerebral areas responsible for the control of anorectal continence. Rectal distension, a situation comparable to the arrival of fecal bolus caused by a high-amplitude propagated contraction (HAPC; Mertz et al., 2000; Hobday et al., 2001; Lotze et al., 2001; Bernstein et al., 2002; Kern and Shaker, 2002; Verne et al., 2003) evokes bilateral activation of the insula, anterior cingulate gyrus, secondary somatosensory cortex and thalamus. Activation of motor areas (M1, Supplementary Motor Area, and cerebellum) occurs exclusively during anal stimulation and is likely a reflex response to rectal distension, with a latency of approximately 6 s (Lotze et al., 2001). Reflex motor activity forms the basis of passive fecal continence, ensuring the containment of fecal bolus within the rectal ampulla (Lotze et al., 2001). Voluntary contraction of the external anal sphincter activates the motor cortex of the supplementary motor area, as well as the primary somatosensory cortex and insula, if repeated (Kern and Shaker, 2002).

Recent studies have also shown co-activation of cortical areas controlling the external anal sphincter and the control areas of the long flexor of the hallux (Rana et al., 2015). This ability to integrate various functions at the cerebral level, such as continence, lower limb movement, and respiration, demonstrates the complexity of the control systems involved in continence at the brain level and seems to be connected to the need to maintain continence under physiological condition (Hodges et al., 2007; Rana et al., 2015).

The overlap control of intestinal and bladder functions is confirmed by the control pathways in the brainstem and spinal cord, as well as the peripheral innervation provided by the pudendal nerve, which is common to both functions (Mackel, 1979).

There is evidence supporting the concept that a pontine defecation center (analogous to the Pontine Micturition Center – PMC) controls the distal colon, rectum, and internal anal sphincter; the external anal sphincter is controlled by the Pontine Continence Center (PCC), which ensures fecal continence (Holstege and Tan, 1987; Rouzade-Dominguez et al., 2003).

The true distinctive element in the control of intestinal function is the ENS, a network composed of approximately half a million neurons spread in the Meissner’s plexus (which regulates intestinal secretions) and the Auerbach’s plexus (responsible for the motor activity of the entire intestine; Furness et al., 2014).

This complex neuronal system is capable of integrating, with excitatory or inhibitory functions, all the reflex activity present in the digestive tract, thereby demonstrating its autonomy from both the central nervous system and the peripheral nervous system. This situation allows us to rightly define it as the “brain in the gut” (Lotze et al., 2001; Lamberti and Biroli, 2020).

The alternation between the filling phase and the emptying phase is under the control of the ENS which ensures propulsion in a proximal-distal direction (but also distal-proximal, a fundamental phenomenon for mixing and nutrient absorption; Bazzocchi et al., 1991); the activation of reflex mechanisms underlying propulsion is determined by the intestinal content, thus making its dimensions crucial (Costa et al., 2015). The propulsion of the food bolus and, in the final segment of the intestine, of the fecal bolus, is ultimately the result of the distension of the intestinal wall (Huizinga et al., 2014). Furthermore, a central feature of intestinal function research is the gut microbiota, which contributes to homeostasis in the human body.

The human body hosts a diverse array of microorganisms forming the microbiome, which plays a crucial role in influencing various physiological processes, including brain health and function. Communication between the brain and the gut microbiota happens through multiple pathways and in a bidirectional manner, involving microbial metabolites, the vagus nerve, the endocrine and the immune systems (Carloni and Rescigno, 2023).

The gut microbiota-brain axis is controlled by the systemic circulation, which is provided with various epithelial and vascular barriers, including: gut-vascular barrier (GVB), blood–brain barrier (BBB), choroid plexus vascular barrier (PVB), blood-cerebrospinal fluid barrier (B-CSF) and intestinal epithelial barrier (IEB; Carloni and Rescigno, 2022).

There is an increased interest in secondary enteric inflammatory bowel disease and dysbiosis, which could result in severe ABI induced neuropathology and neurobehavioral deficits. Microbiome and ABI studies have revealed alterations in the composition of gut microbiota following ABI leading to a state of dysbiosis (Hanscom et al., 2021).

Disruption of the gut barrier integrity, leading to increased permeability and consequent translocation of microbial output into circulation, contributes to systemic immune activation and neuroinflammation (Carloni and Rescigno, 2023). Additionally microbial metabolites, as short chain fatty acids (SCFAs) and neurotransmitter precursors have been implicated in neuroprotection and neuronal repair processes following ABI (Hanscom et al., 2021).

Advancing research in the field of microbiome and acute brain injury requires personalized medicine approaches, identification of microbiome based biomarkers, and well designed clinical trials. Ethical considerations and regulatory frameworks must also be addressed to ensure the safe and responsible application of microbiome based interventions. The microbiome plays a critical role in ABI, influencing pathogenesis, neuroinflammation, and therapeutic responses (Arya and Hu, 2018; Hanscom et al., 2021). Exploring the complex interconnections between microbiome and acute brain injury holds promise for the development of innovative diagnostic-tools and targeted treatments. Continued research efforts are needed to unravel the underlying mechanisms and facilitate the translation of findings into clinical practice, ultimately improving outcomes for individuals affected by ABI. Emerging evidence suggests a relationship between stroke and alterations in the gut microbiota composition (Arya and Hu, 2018; Yamashiro et al., 2021). Dysbiosis may affect stroke outcomes through various mechanisms, including modulation of immune responses, production of metabolites (such as trimethylamine-N-oxide), and disruption of the gut barrier, leading to systemic inflammation. Targeting the microbiome gut-brain axis presents a promising avenue for stroke prevention and management (Yamashiro et al., 2021). CNS damage may result in a loss of voluntary anorectal control (Bharucha and Rao, 2014), with additional social disability for patients (Joan Roach et al., 2000; Camilleri, 2021). Moreover, in patients with ABI, impaired consciousness and memory loss can complicate the assessment of bowel continence (Lim et al., 2012; Emmanuel, 2019). In intensive care units (ICUs), enteral nutrition is associated with diarrhea, one of the most common causes of FI, often a side effect of other treatments (antibiotics, osmolar compounds, and *C. difficile* infection; Reintam Blaser et al., 2015). Drug treatment can also lead to the onset of dysbiosis, which can lead to worse constipation or FI (Weiss and Hennet, 2017). For example, alteration of the gut microbial profile can be caused by using GABA B receptor agonists to treat spasticity (Blackshaw, 2001) or reduction of colon transit time during opioid treatment (Poulsen et al., 2016; Berry et al., 2020).

A broad spectrum of conditions has been extensively studied in NBD epidemiology, including Parkinson’s disease (Stocchi et al., 2000; Awad, 2011), multiple sclerosis (Preziosi et al., 2018; Carotenuto et al., 2021), spinal cord injury (SCI; Emmanuel, 2019; Johns et al., 2021), spina bifida (Emmanuel, 2019), stroke (Harari et al., 2003; Li et al., 2017), and cerebral palsy (Wright et al., 2016).

Neurogenic gut has been extensively studied and investigated in SCI (Stiens et al., 1997; Brading and Ramalingam, 2006). The algorithms and protocols for neurogenic bowel management presented in the literature were aimed at patients with SCI and analyzed intestinal dysfunction according to the reflexia/areflexia of the colon (Stiens et al., 1997; Brading and Ramalingam, 2006). However, in recent years, other factors, such as the microbiota and observations of the enteric system itself, have changed the way neurogenic intestinal problems are treated (Hamilton and Sampson, 2022; Valido et al., 2022).

The assessment of NBD includes descriptions of bowel habits preceding injury or neurological disease, bowel diary, and analysis of current symptoms, including stool consistency (e.g., Bristol stool form scale; O’Donnell et al., 1990) and frequency of bowel movements. In addition, episodes of urgency or flatus/FI, time spent toileting, maneuvers required for evacuation (digital anorectal stimulation, splinting), and use of laxatives or drugs can be assessed.

Rating scales, such as the St. Mark’s incontinence score and Cleveland Clinic constipation score, may be used to quantify symptoms specifically. The precise NBD score has been improved for spinal cord injury and in children with spina bifida (Emmanuel, 2019).

The most common investigation recommended in NBD was the colon transit time (CTT), an abdominal radiograph obtained after ingesting radiopaque markers on a fixed day. Patients with neurological disorders showed delayed transit. Electrophysiological tests and invasive manometry have also been used; their use may be suitable, especially in the presence of past anorectal surgery, obstetrics-gynecology history, and pelvic organ prolapse (POP). Finally, colon imaging and colonoscopy should be carry out in the existence of “red flag” manifestation or patient >50 years (Emmanuel, 2019).

NBD treatment is mainly based on conservative strategies [dietary modifications, laxatives and anti-diarrheal drugs, and trans anal irrigation (TAI)]; however, surgical strategies can also be used, such as antegrade irrigation according to Malone, stoma formation, and sacral neuromodulation (Emmanuel, 2019).

Despite scarce literature, conservative treatment options have been studied in patients with multiple sclerosis and SCI, including conservative measures such as diet (Spinal Cord Medicine Consortium, 1998), antibiotic drugs (Emmanuel, 2010), and TAI (Hultling, 2020) reaching preliminary evidence.

Due to the scarcity of literature and heterogeneity of existing data on ABI NBD (Coggrave et al., 2014; Valbuena Valecillos et al., 2022), a scoping review was planned. The present scoping review aimed to underline the type and entity of evidence regarding bowel dysfunction after brain injury and to present treatment options (except surgery).

The objectives of this study were to understand the number of bowel symptoms in patients with ABI, map assessment tools used in the evaluation of symptoms, and explore the management options for bowel symptoms.

## 2. Methods

This scoping review was conducted according to the PRISMA Extension for Scoping Reviews (PRISMA-ScR; Tricco et al., 2018; Peters et al., 2020); the search protocol was recorded in the Open Science Framework on August 16, 2022.^1^ Reviewers elaborated on search queries following PCC (population, context, and concept) framework as follows:

- Population: patients with bowel dysfunction following ABI, no filter on the trauma mechanism has been added;
- Context: inpatient/outpatient rehabilitation departments;
- Concept: evaluation and treatment of bowel symptoms.

Our research question was developed to better understand the extent of literature about evaluation and treatment of bowel dysfunction in patients with ABI in rehabilitation settings.

Regarding data collection, no time limits were specified for eligible articles; all quantitative study articles, e.g., randomized controlled trials (RCTs), controlled trials without randomization, pre/post studies, quasi-experimental cohorts, and suspended time-series studies, were included. In addition, analytical observational studies, including analytical cross-sectional studies, case–control studies, and retrospective and prospective cohort studies, will be included. Gray literature articles were also considered suitable for review. The Congress Act and extract of the textbooks were excluded.

### 2.1. Inclusion criteria

Studies have been carried out in a rehabilitation setting involving adults diagnosed with bowel dysfunction due to ABI.

### 2.2. Exclusion criteria

Population: studies involving children, spinal cord injury, multiple sclerosis, stroke, Parkinson’s disease and any other conditions determining bowel dysfunction not related to ABI.

Context: home-based rehabilitation setting.

Concept: evaluation/rehabilitation strategies focused on motor/walking function.

### 2.3. Search strategy and data charting

**Figure 1:**
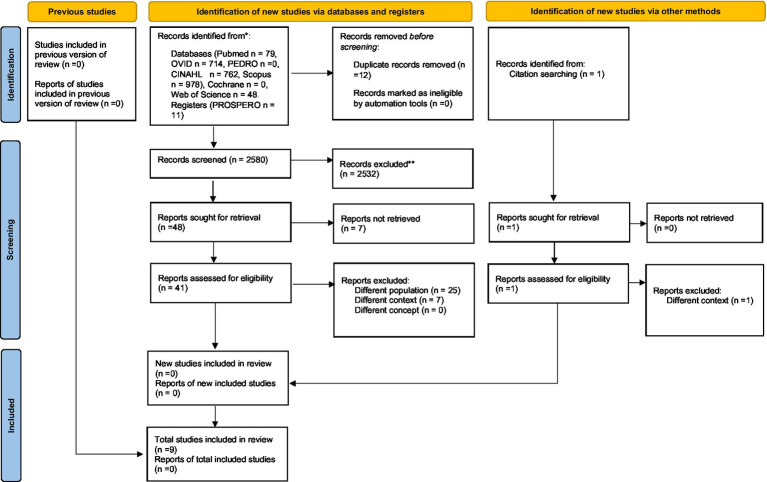
PRISMA 2020 flow diagram of updated systematic reviews which included search of database, register, and other sources. ^*^Consider, if feasible to do so, reporting the number of records identified from each database or register searched (rather than the total number across all databases/registers). ^**^If automation tools were used, indicate how many records were excluded by a human and row many were excluded by automation tools. From Page et al. (2021) (for more information, visit: http://www.prisma-statement.org/).

We searched the following databases Cinhal, Medline (Ovid), Pedro, PubMed, Scopus (Elsevier), Cochrane Library, Web of Science, PROSPERO (NIHR), and sources of unpublished studies/gray literature (open dissertation, clinical trials, Directory of Open Access Journals, and Directory of Open Access Scholarly Resources). For PubMed publications, a specific search string was built, directly derived from PCC, and for other databases, a simple textual search was carried out. The entire search strategy is presented in Table 1. After the removal of duplicates, all data were organized using the Rayyan platform (Ouzzani et al., 2016), an automated online abstraction tool. Two authors (MZ and PS) independently performed the process of evidence screening to obtain at least a double judgment for each article; a first filter by title and abstract was employed. In case of disagreement, a third author (LP) resolved the issue. Includible articles were retrieved in full text for a more in-depth text analysis and the last review round was performed; no critical evaluation was performed on the included articles. A summary data chart was drawn, including all selected articles; for each included article authors and year, sample, intervention and outcome were extracted; the summary of extracted information following the PCC framework was shown in Figure 1.

**Table 1:** PubMed search string.

Domain	Search keywords
Population	Brain injury OR acquired brain injury OR cerebrovascular trauma OR brain injuries, traumatic OR Brain injury OR brain concussion OR Consciousness Disorders OR cognition disorders OR vegetative state OR coma OR unresponsive wakefulness state) AND (neurogenic bowel OR neurogenic bowel dysfunction OR fecal incontinence OR constipation)
Context/Concept	AND (therapeutic use OR physical therapy modalities OR therapy OR Rehabilitation OR assessment, outcome)

## 3. Results

The electronic database search recognized 2,580 plausible studies after elimination of duplication. Following a preparatory examination of keywords, abstracts and titles, 2,432 articles were excluded, and 49 studies were further examined. Although seven studies were not retrieved, 42 studies were checked for eligibility. Based on exclusion criteria, 32 studies were rejected and, finally, 10 full-text articles were included in the review. The publication dates ranged from 2003 to 2022. 1,507 participants were included in the reviewed articles. The most common study model was retrospective 4/10 (40%). A summary of these results is presented in Table 2.

**Table 2:** Result.

Authors and year	Number of patients	Diagnosis	Intervention	Main Outcome
Aadal et al. (2019)	76	Incontinence /Constipation	Laxative	Setting inpatient rehabilitation; On admission the incidence of fecal incontinence is 68 and 32% of fecal constipation. 90% received laxatives in the first month. 35% received combinations of laxatives. After 1 month, the use of laxatives persist in 20% of the patients.
Valbuena Valecillos et al. (2022)	SCI + TBI	Neurogenic Bowel Dysfunction	Suppository, digital stimulation	Setting rehabilitation. Dual diagnosis SCI and TBI from 7 to 74.2%. Rehabilitation goals: regularize fecal evacuation, avoid diarrhea and bowel incontinence, and manage autonomic dysfunction.
Lim et al. (2012)	55	Constipation	Colon transit time (CTT)	Setting inpatient rehabilitation. No correlation between localization brain damage and total CTT or constipation score. CTT of the left colon delay in pontine lesions (*p* < 0.05). The constipation group have increased constipation scores and lower Bristol stool form scale, with delay CTT of total, left, and right colon.
Matsumoto-Miyazaki et al. (2019)	25	Constipation	Acupuncture 2\week for 10 weeks	Setting outpatient rehabilitation. Increase defecation 16.7%, reduction of laxative use.
Kushner and Johnson-Greene (2014)	9	Incontinence	/	Setting inpatient rehabilitation. Improvement of cognitive function follows improvement of continence, maybe due to the prefrontal cortex pathway.
Enevoldsen et al. (2018)	25	Constipation	Laxative occasional	Setting inpatient rehabilitation. Patients with mild to moderate ABI have increase CTT but no related to the heart rate variation (HRV)
Foxx-Orenstein et al. (2003)	1,013	Incontinence	/	Setting inpatient rehabilitation. On admission the incidence of fecal incontinence is 68%, drop out to 12.4% at rehabilitation discharge, and 5.2% at 1-year follow-up
Leary et al. (2006)	238	Incontinence	/	Setting inpatient rehabilitation. On admission 50% of patients reduced bladder/bowel FIM sub scores. At discharge, 36% of patients still had impairment. Although more than 90% of patients set goals on self-care and mobility, only 3.5% patients set goals regarding bladder and bowel function.
New Zealand Guidelines Group (2006)	/	Constipation	/	Recommendations: “verify sufficient fluid intake; use natural laxatives/simple bulk laxatives; perform exercise and standing. Prevent medications reducing gut motility. Increase privacy and comfort during defecation; maintain evacuation routine in a sitting up. If rectum is full, a daily rectal stimulation can be used; if the rectum is empty for 3 days running, the use of an osmotic laxative/stimulant can be evaluated.”
Dourado et al. (2012)	66	Constipation/Incontinence	/	Setting inpatient rehabilitation. Prevalence of constipation 27%, fecal incontinence (FI) 24%. IF associated with motor, communicator and memory impairment.

Regarding the population (patients with ABI with bowel dysfunction), the incidence of FI ranged from 41 to 68% during admission to rehabilitation, dropped to 12–36% at discharge, and reached 5% 1 year after discharge. The incidence of constipation ranges from 32 to 41%, with an index at discharge of approximately 20%. Only one study reported a patient with a double diagnosis of SCI and ABI (Valbuena Valecillos et al., 2022).

Regarding the context (inpatient/outpatient rehabilitation departments), most of the studies involved hospitalized patients, and only one study analyzed outpatient ABI (Matsumoto-Miyazaki et al., 2019).

Regarding the concept (evaluation and treatment of bowel symptoms), the analysis used the Functional Independence Measure (FIM) instrumental subscale (60%; Foxx-Orenstein et al., 2003), followed by the Rome II and III criteria, to assess bowel symptoms in patients with ABI. Instead, to map the assessment tools, only two studies have performed CTT to assess constipation (Lim et al., 2012; Enevoldsen et al., 2018). The CTT study correlates constipation with other neurovegetative parameters such as heart rate variation (HVR), lesion site, and slowest colonic transit area. Finally, to examine management alternatives for bowel symptoms, only one study proposed a trial for constipation using acupuncture (Matsumoto-Miyazaki et al., 2019). Oral laxatives have been proposed as the most common treatment. More than 50% of the articles did not propose specific treatments, focusing on the incidence in the population.

## 4. Discussion

This scoping review distinguished 10 main studies addressing NBD in ABI during rehabilitation. In the management of neurogenic bowel dysfunction, we have to consider the etiopathogenetic mechanisms that contribute to it. There are concurrent alterations in the central nervous system as well as intestinal and microbiota dysfunctions (Carloni and Rescigno, 2023). The gut-brain axis should be understood as a bottom-up interaction: dysbiosis can affect the permeability of the intestinal barrier and, consequently, the blood–brain barrier, leading to processes of cerebral neuroinflammation. However, it should also be understood as a top-down interaction: damage to the CNS system causes oxidative stress and the production of neurotransmitters, which can alter the intestinal bacterial flora (Carloni and Rescigno, 2023). This implies the impossibility of standardizing the extent and type of intestinal dysfunction based on the specific brain localization and the type of damage to the central nervous system. Given the multiple factors involved, management should be comprehensive and encompass both neurological damage and intestinal dysbiosis, as well as nutritional aspects.

### 4.1. Clinical assessment

The most common diagnostic method for constipation diagnosis reported in the literature is the ROME II and III criteria (Drossman and Corazziari, 2000; Drossman, 2016). Table 3 highlights the evolution from ROME II to ROME IV criteria (Drossman and Corazziari, 2000; Longstreth et al., 2006; Drossman, 2016). This method, commonly employed for constipation not associated with neurological issues, is utilized and referenced in the majority of identified articles even for ABI.

**Table 3:** Difference between Rome II vs. Rome III vs. Roma IV (Rome II: Drossman, 1999, Rome III: Longstreth et al., 2006, Rome IV: Drossman, 2016).

Diagnostic Criteria	Rome II (1999) Two or more of the following for at least 12 weeks (not necessary consecutive) in the preceding 12 months:	Rome III (2006) at least two of the following criteria are met for the last 3 months with symptom onset at least 6 months prior to diagnosis	Rome IV (2016) Diagnostic criteria^*^ Must include two or more of the following:^**^
	Straining during (25%) of bowel movement	Straining on >25% of defecations	Straining during more than ¼ (25%) of defecations
	Lumpy or hard stools for >25% of bowel movements	Lumpy or hard stools on >25% of defecations	Lumpy or hard stools (Bristol Stool Form Scale 1–2) more than ¼ (25%) of defecations
	Sensation of incomplete evacuation for >25% of bowel movement	Sensation of incomplete evacuation on >25% of defecations	Sensation of incomplete evacuation more than ¼ (25%) of defecations
	Sensation of anorectal blockage for >25% bowel movement	Sensation of anorectal obstruction/blockage on >25% of defecations	Sensation of anorectal obstruction/blockage more than ¼ (25%) of defecations
	Manual maneuvers to facilitate more than 25% of bowel movement (e.g., digital evacuation, support of the pelvic floor)	Manual maneuvers on >25% of defecations (e.g., digital evacuation, support of the pelvic floor)	Manual maneuvers to facilitate more than ¼ (25%) of defecations (e.g., digital evacuation, support of the pelvic floor)
	Three bowel movement per week	Fewer than 3 defecations per week.	Fewer than three SBM per week
	Loose stools not present	Loose stools must be rarely present without the use of laxatives	Loose stools are rarely present without the use of laxatives
	Insufficient criteria for irritable bowel syndrome met	Insufficient criteria for irritable bowel syndrome	Insufficient criteria for irritable bowel syndrome

* Criteria fulfilled for the last 3 months with symptom onset at least 6 months prior to diagnosis.

** For research studies, patients meeting criteria for opioid-induced constipation (OIC) should not be given a diagnosis of FC because it is difficult to distinguish between opioid side effects and other causes of constipation. However, clinicians recognize that these two conditions may overlap.

The instrument used for the clinical assessment of fecal incontinence, on the other hand, is the FIM scale; FIM bowel management subscale less than 5 was considered FI (Foxx-Orenstein et al., 2003), but this was not constantly used in the various authors analyzed. The most common indirect clinical method to assess stool transit was the Bristol scale (O’Donnell et al., 1990; Lewis and Heaton, 1997), that present high reliability (Chumpitazi et al., 2016).

In addition to being a reliable and routinely used tool, also practical to use in the intestinal diary, the Bristol scale could be a simple indirect indicator of potential dysbiosis, as feces vary in shape and color in cases of dysbiosis (Benno et al., 2019).

### 4.2. Instrumental assessment

CTT was reported as the gold standard for instrumental detection of constipation; however, two protocols, Western (Abrahamsson et al., 1988; Evans et al., 1992) and Asian (Park et al., 2004), were used in clinical practice. Although CTT is a useful tool for constipation, it can only be used in patients without dysphagia due to the shape of the marker. Although CTT was reduced in healthy females (Mugie et al., 2011), no association with sex has been reported in patients with ABI (Dourado et al., 2012; Lim et al., 2012). Despite the absence of an international standardized protocol, CTT has been proposed as a first-level instrumental examination for the assessment of constipation (Arhan et al., 1981). However, future investigations are crucial to test the safety of markers in percutaneous endoscopic gastrostomy to extend examinations in patients with dysphagia.

### 4.3. Management of NBD

The conservative management of NBD in the literature finds limited evidence; indeed, the 2014 Cochrane review (Coggrave et al., 2014) highlights how techniques for bowel management are supported by scarce evidence. Nevertheless, our findings reported only one RCT, which was based on complementary medicine such as acupuncture (Matsumoto-Miyazaki et al., 2019). In this study, 25 patients with chronic disorders of consciousness were treated for constipation using acupuncture sessions twice a week for 10 weeks. There was an increase in defecation frequency from three to 3.5 times a week (*p* < 0.05), with a significant reduction in the use of suppositories. In the study, a single acupuncture point was employed, selected from various points documented in the literature for constipation, known to alter intestinal transit time in an animal study (Iwa et al., 2006). The assessment of constipation improvement relied on clinical parameters, without, however, incorporating intestinal transit time as a measure of efficacy. Moreover, a detailed evaluation of fecal consistency and volume was not conducted.

From a pharmacological perspective, despite the heterogeneity of the population, suppositories and digital stimulation have been reported as constipation treatment options in patients with a double diagnosis of ABI and SCI (Valbuena Valecillos et al., 2022) and these can be regarded as first-line therapeutic choices.

Trans anal irrigation (TAI), as an invasive method, can manage constipation and/or fecal retention and incontinence. Using water to induce the rectal reflex of the colon, TAI can be used in chronic conditions with low side effects (Emmanuel, 2019). TAI is usually well tolerated, can reduce FI, low urinary infection, and improve quality of life (Emmanuel et al., 2016).

The utilization of TAI also enables us to hypothesize significant benefits, particularly considering the operational modes of more recent devices (Bardsley, 2020). Additionally, employing TAI in this phase allows us to address the typical consequences of dysbiosis in these patients (Catanzaro et al., 2019), thus aiming to prevent a worsening of the intestinal neuroinflammatory condition (Sundman et al., 2017; Rice et al., 2019).

### 4.4. Non-conventional therapy

An interesting line of research by Enevoldsen et al. analyzed the correlation between NBD and autonomic dysfunction using heart rate variation (HRV), trying to identify correlations between this and intestinal transit time. However, any correlation between CTT and HVR was shown (Enevoldsen et al., 2018). The Italian ABI minimal protocol (Lavezzi et al., 2022) attempt to analyze autonomic dysfunction in patients with ABI reporting a scale to evaluate the autonomic system with the paroxysmal sympathetic hyperactivity assessment measure (PSHAM; Baguley et al., 2014). It’s interesting to note that autonomic dysfunction is not typically considered in patients with ABI, whereas in patients with SCI, autonomic dysfunction is always taken into account and analyzed, as we can see in the autonomic function after spinal cord injury book (ISAFSCI; Wecht et al., 2021). At the moment, there are no specific targeted treatments for the autonomic nervous system in ABI.

An interesting approach using an osteopathic mesenteric lift to increase bowel movement was proposed for ABI in the ICU (Ward, 2003; Berry et al., 2020). The researchers reported that 77% experienced bowel movements compared to 36% in the control group (*p* = 0.01). This technique has some contraindications, such as severe abdominal pain, infections, metastatic lesions, internal hemorrhage, abdominal aortic aneurysm, recent visceral surgery, and lack of tolerance to treatment (Chila, 2011).

Another original approach was to perform local magnetic stimulation (A-FMS) in a stroke patient with constipation. After the treatment with A-FMS the authors report a 50% reduction in CTT in the left colon and an increase of 50% in the frequency of defecation compared to the sham group (Yun et al., 2019) has been reported.

### 4.5. Consequence of NBD

Fecal incontinence is generally accompanied by the use of laxatives (Aadal et al., 2019), older age (Foxx-Orenstein et al., 2003), memory and communication impairment (Dourado et al., 2012), and damage to the frontal or prefrontal cortex (Foxx-Orenstein et al., 2003). In addition, FI can be used as a marker for the severity of disability (Foxx-Orenstein et al., 2003) and as a predictor of nursing home replacement in the stroke population (Granger et al., 1989). The direct consequences of FI include dermatologic diseases (skin irritation, pressure ulcers, infection) and social problems (reduced activity and participation; Gibson, 1990).

Only one study reported a patient with a double diagnosis of SCI and ABI that increased from 7 to 74% according to different criteria (Valbuena Valecillos et al., 2022). The dissociation between parasympathetic and ENS can contribute to NBD in patients with SCI or traumatic brain injury (TBI; Blanke et al., 2021).

The dysautonomic framework resulting from severe acquired brain injury leads to the disruption of the brain-gut axis, contributing to secondary events related to gastrointestinal disorders, including altered motility, dysbiosis, and increased mucosal permeability. Intestinal disruptions may give rise to heightened systemic inflammation, further exacerbating neuropathological consequences, particularly concerning behavioral symptomatology (Hanscom et al., 2021).

Furthermore, dysbiosis and increased intestinal permeability are linked to heightened blood–brain barrier permeability, leading to a state of neuroinflammation associated with central neurological damage (Carloni and Rescigno, 2022).

Retrospective studies have shown that bowel and urinary management is not well integrated into rehabilitation programs (Leary et al., 2006) and this results in an increase in healthcare and assistance costs for patient management. Indeed an education program during rehabilitation has been suggested to reduce nursing time and as part of a specific rehabilitation program (Cotterill et al., 2018).

### 4.6. Conclusion

NBD is a common consequence after stroke and brain injury (Bracci, 2007; Coggrave et al., 2014). The authors have analyzed the possible mechanisms involved in the pathogenesis of neurogenic bowel dysfunction and the proposed strategies for managing NBD.

This scoping review underlines the need to establish a clearer understanding of potential correlations between the locations of cerebral lesions and the extent of NBD (Turnbull et al., 1999; Kern and Shaker, 2002), particularly given the frequent overlap of constipation and fecal incontinence and their evolution over time (Hakim et al., 2022).

The currently available evidence also highlights how, beyond cerebral localizations, there can be many factors influencing the onset of NBD, such as diet, medication, secondary motor and cognitive difficulties resulting from neurological damage, and alterations in the microbiota; it has also not been possible to identify therapeutic protocols applied early on to prevent the onset of the problem.

The need for a consensus between the rehabilitative and gastroenterological societies on the diagnosis and medical care of bowel dysfunction, particularly in patients with ABI, could be a way to implement patient care and quality of life. In an effort to standardize intestinal management and expand knowledge on the topic the authors advocate the development of an international consensus to deliver bowel management after ABI.

### 4.7. Limitation

This study had several limitations. First, the characteristics of ABI population are unknown in most of the article.

Second, the sample of patients with NBD in ABI has been briefly studied in the literature. Regarding the sample size, most of the samples were from a single US database.

## Data availability statement

The original contributions presented in the study are included in the article/supplementary materials, further inquiries can be directed to the corresponding author.

## Author contributions

GC, MG, GL, and MZ designed the study. MZ, GC, and LP interpreted the data, and wrote the first draft of the manuscript. MZ organized the database and collected the data. MZ, SP, and LP performed the analytical evaluation of articles. All authors contributed to the article and approved the submitted version.

## Footnotes

^1^https://doi.org/10.17605/OSF.IO/NEQMA
